# Supplementary material for: Knowledge and awareness of human papillomavirus infection and human papillomavirus vaccine among Kazakhstani women attending gynecological clinics
Source: PLoS One. 2021 Dec 13;16(12):e0261203. doi: 10.1371/journal.pone.0261203 (PMC8668105; doi:10.1371/journal.pone.0261203)
Supplement: S1 File — (DOCX) [file pone.0261203.s001.docx]

**Survey Protocol**

| **Protocol Title:** | Survey protocol for the study called : **Knowledge and awareness of human papillomavirus infection and human papillomavirus vaccine among Kazakhstani women attending gynecological clinics** |
| --- | --- |
| **Institution/Affiliation:** | Nazarbayev University  <https://nu.edu.kz/> |
| **Principal Investigator Name/Contact Information:** | Gulzhanat Aimagambetova  [gulzhanat.aimagambetova@nu.edu.kz](mailto:gulzhanat.aimagambetova@nu.edu.kz) |
| **Sub Investigator(s) Name/Email:** | Sholpan Akhanova info@keruen-medicus.kz  Natalya Udalova udalovanm@mail.ru  Svetlana Koktova svetlana.koktova@gmail.com  Zhanna Sattarkyzy dr.sattarkyzy@list.ru  Zhuldyz Abakasheva zhyldiz_76@mail.ru  Torgyn Issa torgyn.issa@nu.edu.kz  Aisha Babi [aisha.mukushova@nu.edu.kz](mailto:aisha.mukushova@nu.edu.kz) |
| **Study Contact Person Name/Email:** | Torgyn Issa  [torgyn.issa@nu.edu.kz](mailto:torgyn.issa@nu.edu.kz) |
| **Locations of survey:** | Obstetrics and Gynecology Department, Keruen Medicus Clinic, Almaty 050040, Kazakhstan;  Obstetrics and Gynecology Department, East Kazakhstan Regional Hospital, Oskemen 07000, Kazakhstan;  Regional Perinatal Center, Altynsarin 3, Aktobe 030006, Kazakhstan;  Obstetrics and Gynecology Department, City polyclinic #6, Nur-Sultan 010000, Kazakhstan;  Gynecology Department, Daliya Clinic, Pavlodar 140013, Kazakhstan |
| **Rationale:** | Although cervical cancer remains one of the top causes of cancer-related morbidity and mortality all over the world, there are no currently published studies to assess the knowledge of HPV and cervical cancer in Kazakhstan. |
| **Primary Objectives:** | The aim of this study is to assess the awareness about HPV, the knowledge of HPV as a cause of cervical cancer, and the awareness of HPV vaccination among Kazakhstani women visiting gynecological clinics in five different parts of the country and to identify the factors associated with the awareness of HPV and HPV vaccine, and knowledge of HPV as a major cause of cervical cancer. |
| **Primary Endpoint:** | Outcome variables for this study are the following: awareness of HPV (“Have you ever heard about HPV vaccine?” Yes/No); knowledge of HPV as a major cause of cervical cancer (“HPV infection is the major cause of cervical cancer.” True/False); awareness of HPV vaccine (“There is a vaccine against HPV infection.” True/False). |
| **Study Design:** | Five doctors (Sholpan Akhanova, Natalya Udalova Svetlana Koktova, Zhanna Sattarkyzy, Zhuldyz Abakasheva) will be hired to conduct a survey among  women visiting gynecological clinics. The doctors will have training from research team and from TRAINING AND RESOURCES IN RESEARCH ETHICS EVALUATION. The doctors will be trained how to recruit the participants and how to conduct a survey, and how to store completed questionnaires.  At the end of the appointment doctors will propose their patients to participate in the study.  Before participating in the survey prospective respondents will be provided information about research and doctors will read them verbal consent. Verbal consent will be used in the study.  Particularly for this research verbal consent will be used as way to receive informed consent from a participant. As anonymity of the study participants is our primary concern, verbal consent is deemed as most appropriate. Moreover, no personal information related to any of the patients will be made available to the Investigators at any time before, during or after the study. All the information about the study and participants rights will be stated both orally and on the information letter provided to the participants.  Once the respondent agrees to participate in the study, paper based questionnaires will be given to patients. There will be two types of questionnaires utilized to collect data on patient’s medical gynecological history and awareness and knowledge of HPV, and awareness of HPV vaccination. The first questionnaire will be filled out by doctors and consist of 30 items and include the following patient information: socio-demographic characteristics, lifestyle characteristics, and the history of gynecological diseases. The second questionnaire will be filled out by the respondents. The patient questionnaire will consist of 25 items and include the following information about the patients: socio-demographic characteristics, awareness of cervical cancer and the associated risk factors, awareness of screening for cervical cancer, whether the patient had gone through screening for cervical cancer, awareness of HPV, awareness of risks of HPV, and awareness of HPV vaccine.  Doctors and patients will fill out the questionnaires right after the appointment. For doctors it will take approximately 5 mins to complete the questionnaire. For patients it will take approximately 15 minutes to complete the questionnaire.  To keep anonymity survey respondents will be given IDs. Respondent ID will be written on both questionnaires.  Once completed the respondents will return completed survey to the doctor. The doctor will keep two questionnaires of each respondent in a closed box and only doctor will have access to the questionnaires.  Once doctor will gather 30-40 questionnaires, they will be sent to Nur-Sultan for further analysis and storage. An assigned courier will deliver questionnaire from each city to Nur-Sultan. Questionnaires will be delivered in a sealed folder.  Once questionnaires will be delivered to Nur-Sultan, they will be stored in closed box in PI’s office.  Only complete questionnaires with proper respondent ID will be analyzed for this study.  Data from paper-based questionnaires will be uploaded to computer with the use of Excel software. Once all questionnaires will be uploaded to Excel sheet, data will be imported into STATA16 software and further analyzed. |
| **Inclusion Criteria:** | Women aged between 18 and 70 attending gynecological clinics, and living in one of the cities: Nur-Sultan, Almaty, Aktobe, Oskemen, Pavlodar will be included in the study |
| **Exclusion Criteria:** | Women outside of the age range (18-70) will be excluded from the study |
| **Number of Subjects:** | Approximately 2000 patients will be included in the study. Every doctor will collect around 400 surveys. |
| **Rationale for Number of Subjects:** | The number of patients can be explained by the budget provided to the doctors who will conduct the survey. Each doctor will be paid salary for conducting a survey. |
| **Study Duration:** | The study will be conducted between April 2019 and December 2020. |
| **Risks:** | It is possible that study participants may not:  -feel encouraged to share accurate and honest answers  -feel comfortable providing answers that present themselves in a unfavorable manner  -fully be aware of their reasons for any given answer because of lack of memory and knowledge on the subject |
| **Risk Mitigation:** | In order to avoid the risks outlined above subjects may skip any question they do not wish to answer and/or may withdraw consent from the study at any time without repercussion. |
| **Statistics:** | Statistical analysis will be performed using STATA 16 software. Data analysis will include descriptive statistics consisting of mean values, standard deviations, and frequencies, where applicable. Relationships between categorical variables will be analyzed using Chi-square test with significance value of <0.005. Crude odds' ratio (COR) and adjusted odds ratio (AOR) with 95% corresponding confidence intervals will be calculated in univariate and multivariable logistic regression models. A significance value <0.05 will be used as an indication of association between variables. |
