# Supplementary material for: Knowledge and awareness of human papillomavirus infection and human papillomavirus vaccine among Kazakhstani women attending gynecological clinics
Source: PLoS One. 2021 Dec 13;16(12):e0261203. doi: 10.1371/journal.pone.0261203 (PMC8668105; doi:10.1371/journal.pone.0261203)
Supplement: S2 File — (DOCX) [file pone.0261203.s002.docx]

| Project:________________________________________________________________________________________________________________ | 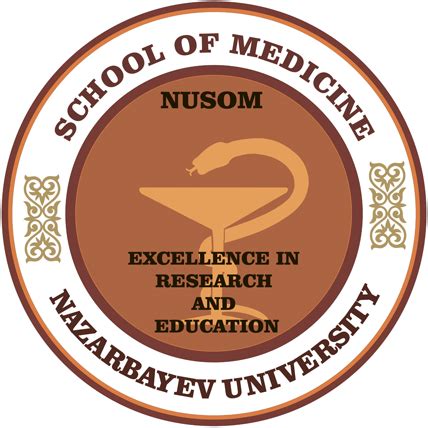 |
| --- | --- |

**Case Number: _____________**

**Enrollment:** ___ / ____ / ___

**Age:**_______ **Ethnicity:** ⬜ Kazakh ⬜ Russian ⬜ Others

**Physician:** ________________ **Institution:** ________________ **Residence:** ___________________

**Telephone:** (home) __________________________ (mobile): _______________________________

**Height (m):** _______**Weight (kg):** **_______**

**Education:** ⬜ Elementary ⬜ Intermediate ⬜ Secondary/College ⬜ University

**Menarche: ____ Menstrual function:** ⬜ Regular ⬜ Irregular**, if irregular** ⬜ Amenorrhea ⬜ Olygomenorrhea ⬜ Polymenorrhea ⬜ Hypermenorrhea ⬜ Hypomenorrhea ⬜ Menorrhagia

**Age at first sexual intercourse**________________**Number of partners**______________________

**Marital status** ⬜ married ⬜ not married ⬜ other_______________________________________

**Gynecological illnesses:**  ⬜ PCOS/PCOD ⬜ Endometriosis ⬜ PID ⬜ Myoma ⬜ Ovarian cyst

**Gyn surgeries**: ⬜ No ⬜ Yes; if yes, Type: _________________________________________

**Patient History:** Gravida: ____ Para: ____Alive children: _______

Abortions: ____Intentional___Sponatneous______ Ectopic pregnancy: ______

**Risk Factors**

**Vaginal Swab:**⬜ Positive ⬜ Negative ⬜ Not done, if positive ⬜ Candida ⬜ Trichomonas v. ⬜ Gonococcus ⬜ BV ⬜ Gram + ⬜ Gram - ⬜ anaerobes

**Infections**

| **HSV** | ⬜ Not sure ⬜ No ⬜ Yes |  | **Syphilis** | ⬜ Not sure ⬜ No ⬜ Yes |
| --- | --- | --- | --- | --- |
| **CMV** | ⬜ Not sure ⬜ No ⬜ Yes |  | **Mycoplasma** | ⬜ Not sure ⬜ No ⬜ Yes |
| **Chlamydia** | ⬜ Not sure ⬜ No ⬜ Yes |  | **Ureaplasma** | ⬜ Not sure ⬜ No ⬜ Yes |

**History of Pelvic Inflammatory Disease:** ⬜ Negative ⬜ Positive, **if positive**, please add detailes________________________________________________________________________________________________________________________________________________________________

**Contraceptives:** ⬜ No ⬜ Yes If Yes, Type: _______________________ Duration: _______

**Smoking:** ⬜ No ⬜ Yes 🞎 Former; Frequency: ____/day **Alcohol Consumption:** ⬜ No ⬜ Yes

**Pap Smear:** ⬜ Normal or **CIN grade:** ⬜ **1** ⬜ **2** ⬜ **3, or** ⬜ **NILM** ⬜ **HSIL** ⬜ **LSIL**

**Management:** ⬜ Conservative, ⬜ Surgical (conization, etc.), ⬜ Other

**History of Cervical cancer**: ⬜ Negative ⬜ Positive, **if positive** please add detailes________________________________________________________________________________________________________________________________________________________________

**Family history**

**Family history of cervical cancer:** ⬜ Negative ⬜ Positive, **if positive** please add detailes________________________________________________________________________________________________________________________________________________________________

**Family history of ovarian cancer**: ⬜ Negative ⬜ Positive, **if positive** please add detailes________________________________________________________________________________________________________________________________________________________________

**Family history of breast cancer:** ⬜ Negative ⬜ Positive, **if positive** please add detailes________________________________________________________________________________________________________________________________________________________________

**History of any other cancer localization:**   No  Yes For parents or siblings:  No  Yes
