# Supplementary material for: Knowledge and awareness of human papillomavirus infection and human papillomavirus vaccine among Kazakhstani women attending gynecological clinics
Source: PLoS One. 2021 Dec 13;16(12):e0261203. doi: 10.1371/journal.pone.0261203 (PMC8668105; doi:10.1371/journal.pone.0261203)
Supplement: S3 File — (DOCX) [file pone.0261203.s003.docx]

**Questionnaire**

Date /___ /___ /__ __ __

Participant №__________

1. **How old are you?** **(write in)** _______________
2. **Your highest obtained degree?**

- Primary school
- Secondary school
- High school
- Technical and vocational education
- Bachelor degree
- Master’s or PhD degree

1. **What is your place of residence?**

- Astana
- Almaty
- Aktobe
- Oskemen
- Pavlodar
- Other (write in)_________________

1. **Your marital status?**

- Single
- Married
- In committed relationship
- Divorced
- Widowed
- Other (specify)__________________

1. **How many children do you have?**

- 0
- 1
- 2
- 3 or more

1. **How many deliveries did you have?**

- 0
- 1
- 2
- 3 or more

1. **What is your monthly family income (tenge)**?

- Less or equal to 100 000
- 101 000 - 200 000
- 201 000 – 300 000
- 301 000 – 400 000
- 401 000 or more

1. **What do you know about cervical cancer?**

- Not caused by infection and cannot be prevented
- Caused by viral infection and can be prevented
- Caused by viral infection and cannot be prevented
- Nothing

1. **Which of the following might increase the risk of cervical cancer? (can choose several answers)**

- A failure to use condoms
- Early age of first sexual intercourse
- Smoking
- High number of sexual partners
- Other (specify)_________________

1. **What is an oncocytology test (Pap smear test)?**

- Scraping to look for abnormal cells
- Speculum in the vagina
- Treatment for cancer
- Test for a sexually transmitted disease
- Don’t know

1. **What is the recommended frequency for oncocytology (Pap smear) test?**

- Annually
- Every 3–5 years
- Every 10 years
- Don’t know

1. **What do you think an abnormal oncocytology (Pap smear) result might mean?**

- Abnormal, precancerous cells
- Presence of cancer
- Presence of infection
- Other (specify)______________
- Don’t know

1. **Is free screening program for cervical cancer (Pap smear test) available in Kazakhstan?**

- Yes
- No
- Not sure

1. **If you answered *yes*, what sources have you relied on for information on free screening?**

- General practitioner
- Gynecologist
- Practice nurse
- Other health professionals
- Television
- Magazines/books
- Internet
- Educational settings (School, University)
- Family members
- Friends and peers
- Other (specify)_________________

1. **Have you ever undergone screening for cervical cancer (Pap smear test) in the past?**

- Yes, I used free screening
- Yes, I used screening on a paid basis
- No, I didn’t use any screening program

1. **If you answered *no*, why?**

____________________________________________________________

1. **Have you ever heard of Human Papilloma Virus (HPV) infection?**

- Yes
- No *(Survey is finished)*

1. **If you answered *yes,* what sources have you relied on for information on HPV infection?**

- General practitioner
- Gynecologist
- Practice nurse
- Other health professionals
- Television
- Magazines/books
- Internet
- Educational settings (School, University
- Family members
- Friends and peers
- Other (specify)_________________

1. **How do you think HPV infection is contracted?**

- Sexually
- Air borne route
- Fecal-oral route
- Blood borne route
- Don’t know

1. **Which of the following increase the risk of contracting HPV infection?** **(can choose several answers)**

- Early puberty
- Multiple sexual partners
- If partner has had multiple sexual partners
- Early sexual activity
- Failure to use condoms
- Other

1. **HPV is an infection which affects:**

- Only or mainly men
- Only or mainly women
- Both men and women
- Don’t know

1. **HPV infection is the major cause of cervical cancer**

- True
- False
- Don’t know

1. **The oral contraceptive pills protect against HPV infection**

- True
- False
- Not sure

1. **Condoms protect against HPV infection**

- True
- False
- Not sure

1. **There is a vaccine against HPV infection**

- True
- False
- Not sure
