# Supplementary material for: Knowledge and awareness of human papillomavirus infection and human papillomavirus vaccine among Kazakhstani women attending gynecological clinics
Source: PLoS One. 2021 Dec 13;16(12):e0261203. doi: 10.1371/journal.pone.0261203 (PMC8668105; doi:10.1371/journal.pone.0261203)

PLOS ONE

Knowledge and awareness of human papillomavirus infection and human papillomavirus vaccine among Kazakhstani women attending gynecological clinics

--Manuscript Draft--

**Manuscript Number:**	PONE-D-21-11756
**Article Type:**	Research Article
**Full Title:**	Knowledge and awareness of human papillomavirus infection and human papillomavirus vaccine among Kazakhstani women attending gynecological clinics
**Short Title:**	Short title: Knowledge and awareness of HPV and HPV vaccine among women
**Corresponding Author:**	Gulzhanat Aimagambetova<br>Nazarbayev University School of Medicine<br>Nur-Sultan, Akmola region KAZAKHSTAN
**Keywords:**	cervical cancer; HPV; HPV knowledge; HPV awareness; Kazakhstan
**Abstract:**	Cervical cancer remains one of the top causes of cancer-related morbidity and mortality all over the world. However, currently there are no published studies to assess the knowledge of HPV and cervical cancer at the country level. This study aimed to assess the awareness about HPV, the knowledge of HPV as a cause of cervical cancer, and the awareness of HPV vaccination among Kazakhstani women visiting gynecological clinics in five different parts of the country and to identify the factors associated with the awareness of HPV and HPV vaccine, and knowledge of HPV as a major cause of cervical cancer.This was a cross-sectional survey-based study with 2,281 participants among women attending gynecological clinics aged between 18-70 years who were administered paper-based questionnaires. Data analysis included descriptive statistics consisting of mean values, standard deviations, and frequencies, where applicable. Differences in categorical variables between groups were analyzed using Chi-square test with a significance value of <0.005. Crude odds' ratio (OR) and adjusted odds ratio (AOR) with 95% corresponding confidence intervals were calculated in regression analysis using univariate and multivariable logistic regression models. The mean age of participants was 36.33±10.09 years. More than half (53.43%) of the participants had been screened for cervical cancer. Among those who were aware of HPV, 46.36% knew that HPV causes cervical cancer and 51.53% were aware of HPV vaccine. The key factors related to outcome variables were age, ethnicity, education, family, number of deliveries, and menarche. From a subgroup analysis, results from HPV test and Pap smear test were factors related to dependent variables such as awareness of HPV and awareness of HPV vaccination.
**Order of Authors:**	Torgyn Issa
	Aisha Babi
	Alpamys Issanov
	Ainur Akilzhanova
	Kadisha Nurgaliyeva
	Zauresh Abugalieva
	Azliyati Azizan
	Saleem A. Khan
	Chee Kai Chan
	Raushan Alibekova
	Gulzhanat Aimagambetova
**Additional Information:**	
**Question**	**Response**
**Financial Disclosure**	This study was supported by the Faculty Development Research Grant Program 2019-

Enter a financial disclosure statement that describes the sources of funding for the work included in this submission. Review the submission guidelines for detailed requirements. View published research articles from *PLOS ONE* for specific examples.<br><br>This statement is required for submission and **will appear in the published article** if the submission is accepted. Please make sure it is accurate.<br><br>**Unfunded studies**<br>Enter: *The author(s) received no specific funding for this work.*<br><br>**Funded studies**<br>Enter a statement with the following details:<br>• Initials of the authors who received each award<br>• Grant numbers awarded to each author<br>• The full name of each funder<br>• URL of each funder website<br>• Did the sponsors or funders play any role in the study design, data collection and analysis, decision to publish, or preparation of the manuscript?<br>• **NO** - Include this sentence at the end of your statement: *The funders had no role in study design, data collection and analysis, decision to publish, or preparation of the manuscript.*<br>• **YES** - Specify the role(s) played.<br><br>* typeset	2021 (Funder Project Reference: 110119FD4528, title: A molecular epidemiological study to determine the prevalence of oncogenic HPV strains for CC prevention in Kazakhstan). The funder had no role in study design, data collection and analysis, decision to publish, or preparation of the manuscript. GA. is a PI of the project.
**Competing Interests**<br><br>Use the instructions below to enter a competing interest statement for this submission. On behalf of all authors, disclose any competing interests that could be perceived to bias this work—acknowledging all financial support and any other relevant financial or non-financial competing interests.<br><br>This statement is required for submission and **will appear in the published article** if	The authors have declared that no competing interests exist.

the submission is accepted. Please make sure it is accurate and that any funding sources listed in your Funding Information later in the submission form are also declared in your Financial Disclosure statement.<br><br>View published research articles from *PLOS ONE* for specific examples.<br><br>**NO authors have competing interests**<br><br>Enter: *The authors have declared that no competing interests exist.*<br><br>**Authors with competing interests**<br><br>Enter competing interest details beginning with this statement:<br><br>*I have read the journal's policy and the authors of this manuscript have the following competing interests: [insert competing interests here]*<br><br>* typeset	
**Ethics Statement**<br><br>Enter an ethics statement for this submission. This statement is required if the study involved:<br><br>• Human participants<br>• Human specimens or tissue<br>• Vertebrate animals or cephalopods<br>• Vertebrate embryos or tissues<br>• Field research<br><br>Write "N/A" if the submission does not require an ethics statement.<br><br>General guidance is provided below. Consult the submission guidelines for detailed instructions. **Make sure that all information entered here is included in the Methods section of the manuscript.**	This study design and the content of the questionnaires were approved by the Nazarbayev University Institutional Research Ethics Committee (NU IREC), protocol number 146/4042019, date of approval 23/04/2019. Verbal informed consent was received from all participants.

Powered by Editorial Manager® and ProduXion Manager® from Aries Systems Corporation

**Format for specific study types**<br><br>**Human Subject Research (involving human participants and/or tissue)**<br>• Give the name of the institutional review board or ethics committee that approved the study<br>• Include the approval number and/or a statement indicating approval of this research<br>• Indicate the form of consent obtained (written/oral) or the reason that consent was not obtained (e.g. the data were analyzed anonymously)<br><br>**Animal Research (involving vertebrate animals, embryos or tissues)**<br>• Provide the name of the Institutional Animal Care and Use Committee (IACUC) or other relevant ethics board that reviewed the study protocol, and indicate whether they approved this research or granted a formal waiver of ethical approval<br>• Include an approval number if one was obtained<br>• If the study involved *non-human primates*, add *additional details* about animal welfare and steps taken to ameliorate suffering<br>• If anesthesia, euthanasia, or any kind of animal sacrifice is part of the study, include briefly which substances and/or methods were applied<br><br>**Field Research**<br><br>Include the following details if this study involves the collection of plant, animal, or other materials from a natural setting:<br>• Field permit number<br>• Name of the institution or relevant body that granted permission	
**Data Availability**<br><br>Authors are required to make all data underlying the findings described fully available, without restriction, and from the time of publication. PLOS allows rare exceptions to address legal and ethical concerns. See the PLOS Data Policy and FAQ for detailed information.	Yes - all data are fully available without restriction

Powered by Editorial Manager® and ProduXion Manager® from Aries Systems Corporation

A Data Availability Statement describing where the data can be found is required at submission. Your answers to this question constitute the Data Availability Statement and **will be published in the article**, if accepted.<br><br>**Important:** Stating 'data available on request from the author' is not sufficient. If your data are only available upon request, select 'No' for the first question and explain your exceptional situation in the text box.<br><br>Do the authors confirm that all data underlying the findings described in their manuscript are fully available without restriction?	
**Describe where the data may be found in full sentences. If you are copying our sample text, replace any instances of XXX with the appropriate details.**<br><br>• If the data are **held or will be held in a public repository**, include URLs, accession numbers or DOIs. If this information will only be available after acceptance, indicate this by ticking the box below. For example: *All XXX files are available from the XXX database (accession number(s) XXX, XXX.)*.<br>• If the data are all contained **within the manuscript and/or Supporting Information files**, enter the following: *All relevant data are within the manuscript and its Supporting Information files.*<br>• If neither of these applies but you are able to provide **details of access elsewhere**, with or without limitations, please do so. For example:<br><br>*Data cannot be shared publicly because of [XXX]. Data are available from the XXX Institutional Data Access / Ethics Committee (contact via XXX) for researchers who meet the criteria for access to confidential data.*<br><br>*The data underlying the results presented in the study are available from (include the name of the third party*	The study questionnaires and raw data are available via the link: https://zenodo.org/record/4600664#.YHBChs9xfIV

and contact information or URL).<br>• This text is appropriate if the data are owned by a third party and authors do not have permission to share the data.<br><br>* typeset	
Additional data availability information:	

Manuscript

Click here to access/download;Manuscript;plos one_manuscript.docx

Full title: **Knowledge and awareness of human papillomavirus infection and human papillomavirus vaccine among Kazakhstani women attending gynecological clinics**

Short title: Knowledge and awareness of HPV and HPV vaccine among women

Torgyn Issa[1¶], Aisha Babi[1¶], Alpamys Issanov[1¶], Ainur Akilzhanova[2], Kadisha Nurgaliyeva[3], Zauresh Abugalieva[3], Azliyati Azizan[1,4], Saleem A. Khan[5], Chee Kai Chan[1,6], Raushan Alibekova[1], Gulzhanat Aimagambetova[1*]

[1] School of Medicine, Nazarbayev University, Nur-Sultan, Kazakhstan

[2] National Laboratory of Astana, Nazarbayev University, Nur-Sultan, Kazakhstan

[3] Republican Diagnostic Center, University Medical Center, Nur-Sultan, Kazakhstan

[4] College of Osteopathic Medicine, Touro University Nevada, Henderson, Nevada, USA

[5] School of Medicine, University of Pittsburgh, Pennsylvania, USA

[6] College of Science and Technology, Wenzhou-Kean University, Wenzhou, China

*Corresponding Author:

Email address: gulzhanat.aimagambetova@nu.edu.kz: Tel.: +77017529301

[¶]These authors contributed equally to this work

Abstract

Cervical cancer remains one of the top causes of cancer-related morbidity and mortality all over the world. However, currently there are no published studies to assess the knowledge of HPV and cervical cancer at the country level. This study aimed to assess the awareness about HPV, the knowledge of HPV as a cause of cervical cancer, and the awareness of HPV vaccination among Kazakhstani women visiting gynecological clinics in five different parts of the country and to identify the factors associated with the awareness of HPV and HPV vaccine, and knowledge of HPV as a major cause of cervical cancer.

This was a cross-sectional survey-based study with 2,281 participants among women attending gynecological clinics aged between 18-70 years who were administered paper-based questionnaires. Data analysis included descriptive statistics consisting of mean values, standard deviations, and frequencies, where applicable. Differences in categorical variables between groups were analyzed using Chi-square test with a significance value of <0.005. Crude odds' ratio (OR) and adjusted odds ratio (AOR) with 95% corresponding confidence intervals were calculated in regression analysis using univariate and multivariable logistic regression models.

The mean age of participants was 36.33±10.09 years. More than half (53.43%) of the participants had been screened for cervical cancer. Among those who were aware of HPV, 46.36% knew that HPV causes cervical cancer and 51.53% were aware of HPV vaccine. The key factors related to outcome variables were age, ethnicity, education, family, number of deliveries, and menarche. From a subgroup analysis, results from HPV test and Pap smear test were factors related to dependent variables such as awareness of HPV and awareness of HPV vaccination.

Introduction

Human papillomavirus (HPV) is one of the most common sexually transmitted infections in the world [1]. There is an estimate that 80% of people will acquire HPV in their lifetime [2] . The prevalence of HPV was reported to vary from 7 to 14% in the general population [3]. Out of numerous types, HPV 16 and HPV 18 are causally associated with 70% of cervical cancer cases and precancerous cervical lesions [1].

Cervical cancer is the fourth most common cancer among women worldwide [4]. In 2018, there were 570,000 cases of cervical cancer and around 311,000 deaths from cervical cancer [5]. Moreover, according to the World Health Organization (WHO), by 2030 it is expected that the number of new cervical cancer cases will reach 700,000. At the same time, the absolute number of deaths from cervical cancer will reach 400,000 [1]. Therefore, based on such a significant annual increase in number of cases and deaths, cervical cancer represents a major global public health challenge.

In 2020, WHO launched a global program to eliminate cervical cancer that was announced earlier, in 2018 [6]. The major steps highlighted in WHO's Global Strategy to Accelerate the Elimination of Cervical Cancer were vaccination, screening, and treatment [7]. In order to reduce cases of cervical cancer, WHO has assigned all countries in the world to meet the requirements. First, 90% of young females should be vaccinated against HPV before the age of 15 [6]. Secondly, 70% of women should be screened for cervical cancer using high-performance test by the age of 35, and repeatedly by the age of 45 [1]. According to the third requirement of the strategy, 90% of women who are diagnosed with cervical neoplastic lesions should receive appropriate treatment [6].

Kazakhstan is a Central Asian country with a land area of more than 2,724 thousand km^2 and is the ninth largest country in the world. The population of the country is estimated at almost 19 million. After obtaining independence from the Soviet Union, Kazakhstan has undergone a drastic transformation in the healthcare system [8]. In Kazakhstan, as of today, there are no published studies on the prevalence of HPV at the country level. However, some data on the HPV prevalence is available from regional and pilot studies [9,10]. However, the results of these studies cannot be generalized for the whole country as it has a large territory with diverse population.

There is also a lack of local research to determine the statistics of cervical cancer. According to the HPV Information Centre report, cervical cancer ranks as the second leading cause of cancer among women and cancer-related death in Kazakhstan. Cervical cancer remains as the second leading cause of female cancer and cancer-related deaths in Kazakhstani women with over 1,700 new cervical cancer cases diagnosed annually [11,12]. In comparison with global estimates, cervical cancer statistics remain high in Kazakhstan.

Cervical cytology remains the main screening method for cervical cancer in Kazakhstan. From 2017 until current days, the screening approach is the same using the Pap test, but the screening age group was expanded to include all women between the age of 30-70 years [13]. Although plans for 2019-2020 included HPV genotyping as a part of the cervical cancer screening program [13], HPV testing is only available on a commercial basis. Despite the free nature of the cervical cancer screening program in Kazakhstan, screening coverage remains low. Between 2008 and 2016, 4,460,320 women from the whole country had undergone screening for cervical cancer[14]. During these nine years, there was a 32% decrease in the number of women who underwent screening[14].

HPV vaccines (Gardasil and Cervarix) were introduced as a pilot program in four regions of Kazakhstan in 2013. However, the absence of an HPV vaccination communication plan, the media's negative coverage of these cases, combined with insufficient training of health care workers, had a negative impact on the public's willingness to receive the vaccine. The vaccination coverage was suspended in 2015 while there are more than 6 million women still at risk of cervical cancer in the country [11,15].

The success and benefit of control and prevention of cervical cancer largely depend on the level of awareness and knowledge about different aspects of the disease and the vaccine. Such low coverage of free cancer screening program and the lack of free HPV testing could be related to the lack of knowledge in the general population regarding HPV and cervical cancer. It is important to mention that WHO's Global Strategy to Accelerate the Elimination of Cervical Cancer not only addresses the importance of cervical cancer screening but also the importance of HPV vaccination as a prevention strategy for this disease. Moreover, with the plan for the HPV vaccine introduction on the national level in Kazakhstan [13], the education and awareness among the general population becomes even more significant for the success of the program. Knowledge and understanding of behavioral perception towards HPV vaccination are crucial for the development of an effective health policy that will support HPV vaccination among the population [16].

This study aims to assess the awareness of HPV, the knowledge of HPV as a cause of cervical cancer, and the awareness of HPV vaccination among Kazakhstani women visiting gynecological clinics in five different parts of the country. Moreover, the purpose of this study is to identify the factors, such as social and demographic characteristics, and gynecological health characteristics of the participants, associated with the awareness of HPV and HPV vaccine, and knowledge of HPV as a major cause of cervical cancer.

Materials and Methods

Study participants and sample collection

A prospective cross-sectional study among women from five cities of central (Nur-Sultan, the capital city), southern (Almaty), western (Aktobe), northern (Pavlodar), and eastern (Oskemen) parts of Kazakhstan was conducted from May 25, 2019, until December 2020. Women aged between 18 and 70 attending gynecological clinics were recruited to the study by convenience sampling method.

Study instrument

There were two types of questionnaires utilized to collect data on patients medical gynecological history and awareness and knowledge of HPV, and awareness of HPV vaccination. The first questionnaire was filled out by doctors and consisted of 30 items and included the following patient information: socio-demographic characteristics, lifestyle characteristics, and the history of gynecological diseases.

The second questionnaire that was filled out by the participants was adapted from previous studies [17,18]. The patient questionnaire consisted of 25 items and included the following information about the patients: socio-demographic characteristics, awareness of cervical cancer and the associated risk factors, awareness of screening for cervical cancer, whether the patient had gone through screening for cervical cancer, awareness of HPV, awareness of risks of HPV, and awareness of HPV vaccine. Only those who were aware of HPV continued to answer the questions about knowledge of HPV and awareness of HPV vaccine. The survey was conducted in Kazakh and Russian languages depending on the preferences of the participants.

Sample collection and genotyping

All the study participants had Papanicolaou (Pap smear) testing done. The cervical swabs were used as a source for HPV genotyping using AmpliSens kit that identifies 14 high-risk HPV types : 16, 18, 31, 33, 35, 39, 45, 51, 52, 56, 58, 59, 66, 68. The swab samples were collected into 1.5 mL Eppendorf tubes by gynecologists using cytobrush and were transported and stored in a frozen state (-20°C) until needed for DNA extraction procedure. DNA was extracted using the Wizard® Genomic DNA Purification Kit according to the manufacturer's protocol. Real-time PCR analysis was performed using the CFX 96 Real-Time PCR detection system from the Bio-Rad Laboratories. Each PCR run had positive, negative and internal controls, as per the manufacturer's instructions. Genotyping results were divided into two outcomes: positive - having any of the 14 high-risk HPV types and negative – does not have any of these HPV types.

Study variables

Independent variables were socio-economic and demographic characteristics (age, ethnicity (Kazakh, Russian and other ethnicities); city of residence) of the participants. Also, information on marital status (not single - married, in committed relationship; single - single, widowed, divorced), and family (number of children, history of delivery, history of abortion) characteristics of participants were collected. Gynecological health (age at the start of sex life, menarche, result of cervical cytology test by Pap smear, result of HPV genotyping test) were collected.

Outcome variables for this study were the following: awareness of HPV ("Have you ever heard about HPV vaccine?" Yes/No); knowledge of HPV as a major cause of cervical cancer ("HPV infection is the major cause of cervical cancer." True/False); awareness of HPV vaccine ("There is a vaccine against HPV infection." True/False).

Ethical considerations

The ethical approval to conduct this study was given by the Institutional Research Ethics Committee of Nazarbayev University (NU-IREC) on April 23, 2019 (IREC decision number: 146/4042019). Study participants were informed about the risks, benefits, goals, and methods of the study. After receiving verbal consent, study participants responded to the survey questions.

Particularly for this research we used verbal consent as way to receive informed consent from a participant. As anonymity of the study participants was our primary concern, verbal consent was deemed as most appropriate. Moreover, no personal information related to any of the patients was made available to the Investigators at any time before, during or after the study. All the information about the study and participants rights was stated both orally and on the information letter provided to the participants.

It is important to mention the cultural context. Verbal consent was prioritized as Kazakhstan is a post-Soviet country where people still are not comfortable with signing documents that are similar to a contract. Moreover, general population tends to have low trust towards interviews and researchers. Such low trust to the confidentiality of research is mostly common among elder generation, and our study recruited participants until the age of 70.

Verbal consent used for this study included all the necessary components for the informed consent. First of all the doctor explained the title, research itself and the major goals of the research. Secondly the informed consent included detailed information on how long the participation will take time and what kind of questions are included. Thirdly the verbal consent included the information on patient confidentiality, patient risks and rewards. Lastly the informed consent included the contacts of the Principal Investigator of the research; in case patients had any questions.

Data analysis

Statistical analysis was performed using STATA 16 [19]. Data analysis included descriptive statistics consisting of mean values, standard deviations, and frequencies, where applicable. Relationships between categorical variables were analyzed using Chi-square test with significance value of <0.005. Crude odds' ratio (COR) and adjusted odds ratio (AOR) with 95% corresponding confidence intervals were calculated in univariate and multivariable logistic regression models. A significance value <0.05 was used as an indication of association between variables.

Results

Participant characteristics

The total number of participants recruited for the study was 2,281. The descriptive characteristics of the study participants are shown in Table 1. Study participants were diverse in terms of age, ethnicity, educational level, city, family, age of the first menarche, age during the first intercourse, number of living children, and number of deliveries, number of abortions, Pap test result, and HPV status. Study participants had a mean age of 36.33±10.09 with a range of 18-70 years old. The majority of the participants were aged between 26 and 35 (39.45%). More than three quarters (75.35%) of women belonged to Kazakh ethnicity. Almost half of women (47.71%) had undergraduate and/or graduate university degrees. Approximately, an equal number of participants were recruited from five cities, with Nur-Sultan having the least number of participants (15.49%). The majority of the participants were married or in a relationship (81.35%). The mean age of menarche of the participants was 13.60±1.41, most (60.95%) had menarche between 13 and 15. The mean age of the first intercourse was 20.47±2.97, and the

majority had sex after age of 18 years (90.28%). More than three quarters of women have had one or more children and deliveries. More than half (51.26%) of the respondents had one or more abortions in their life. The majority of women (92.45%) had normal Pap test results. More than half of the participants had negative results of the HPV genotyping test.

Table 1. Demographic and clinical characteristics of women visiting gynecological clinics, (N=2281)

**Variables**	**Total N=2281, %**
**Age,** mean 36.33±10.09	
**18-25**	13.63
**26-35**	39.45
**36-45**	27.92
**46+**	19.00
**Ethnicity**	
**Kazakh**	75.37
**Russian**	18.47
**Other**	6.16
**Education**	
**Unfinished/finished school**	75.37
**College**	18.47
**University**	6.16
**City**	
**Nur-Sultan**	15.49
**Almaty**	21.78
**Aktobe**	21.56
**Oskemen**	20.33
**Pavlodar**	20.85
**Family**	
**Single**	18.65
**Not single**	81.35
**Age of menarche**	
**<13**	15.3
**13-15**	60.95
**>15**	23.75
**Age of first intercourse,** mean 20.47±2.97	
**< 18**	9.72
**=>18**	90.28
**Number of alive children**	

**0**	20.06
**≥1**	79.94
**Number of deliveries**	
**0**	19.36
**≥1**	80.64
**Number of abortions**	
**0**	48.24
**≥1**	51.76
**Pap test status**	
**Normal**	92.45
**Abnormal**	7.55
**HPV status**	
**Negative**	61.42
**Positive**	38.58

HPV awareness

Almost half of the respondents (53.43%) were informed about HPV as you can see in Table 2. Bivariate analysis using the chi-square test showed that HPV awareness was statistically significantly associated with age, ethnicity, education level, city, family groups, menarche groups, and number of deliveries, Pap test results, and HPV status.

Table 2. Awareness of HPV among women visiting gynecological clinics, (N=2281)

**Variables**	**Aware of HPV N = 1215; 53.43% %, p value**	**OR (95% CI) N=2273**	**AOR (95% CI)* N=2273**		**AOR subgroup (95% CI)** N=729**
**Age,** mean 36.33±10.09					
			No deliveries	One or more deliveries	
**18-25**	51.61	1	1	1	1
**26-35**	50.84	0.96 (0.75-1.26)	1.19 (0.77-1.85)	0.96 (0.67-1.37)	1.01 (0.60-1.71)
**36-45**	58.11	1.30 (0.98-1.71)	0.73 (0.39-1.34)	1.55 (1.07-2.23)	1.37 (0.78-2.42)
**46+**	53.24	1.07 (0.79-1.43)	1.44 (0.62-3.33)	1.28 (0.87-1.87)	1.03 (0.55-1.92)
	p-value =				

	0.038			
**Ethnicity**				
**Kazakh**	49.07	1	1	1
**Russian**	65.95	2.01 (1.61-2.51)	1.98 (1.57-2.49)	2.11 (1.45-3.08)
**Other**	69.29	2.34 (1.61-3.39)	2.24 (1.54-3.27)	2.07 (1.11-3.87)
	p-value = 0.000			
**Education**				
**Unfinished/finished school**	45.64	1	1	1
**College**	49.49	1.17 (0.91-1.49)	1.24 (0.97-1.60)	0.90(0.56-1.45)
**University**	59.17	1.73 (1.37-2.17)	1.81 (1.43-2.30)	1.28 (0.83-1.97)
	p-value= 0.000			
**Age of menarche,** mean 13.60±1.41				
**<13**	61.78	1	1	1
**13-15**	54.69	0.75 (0.59-0.95)	0.82 (0.64-1.04)	0.91 (0.57-1.47)
**>15**	44.81	0.50 (0.38-0.66)	0.55 (0.42-0.74)	0.73 (0.42-1.26)
	p-value= 0.000			
**Number of deliveries**				
**0**	58.18	1	1	1
**≥1**	52.26	0.78 (0.64-1.68)	0.78 (0.49-1.24)	0.82 (0.54-1.25)
	p-value = 0.025			
**Pap test status**				
**Normal**	56.43	1		1
**Abnormal**	39.57	0.51 (0.36-0.72)		0.47 (0.26-0.88)
	p-value= 0.000			
**HPV status**				
**Negative**	42.92	1		1
**Positive**	55.29	1.64 (1.23-2.21)		1.63 (1.19-2.24)
	p-value = 0.001			

*Adjusted for age, ethnicity, education, age of menarche, number of deliveries
**Adjusted for age, ethnicity, education, age of menarche, number of deliveries, Pap test status, HPV status

In the multivariable logistic model, the following factors were positively associated with HPV awareness: age group of 46+ (AOR=1.44.; CI:0.62-3.33, referent=age group of 18-25), Other ethnic group (AOR=2.24; CI:1.54-3.27,), university degree (AOR=1.81; CI:1.43-2.30,), having family or being in a relationship (AOR=1.01; CI:0.68-1.51). Factors negatively associated with HPV awareness were the following: age group of 36-45 (AOR=0.73.; CI: 0.39-1.34, referent=age group of 18-25), menarche after 15 (AOR=0.55; CI: 0.42-0.74,) and having one or more deliveries (AOR=0.78; CI: 0.49-1.24, referent=having no delivery[illegible] (Table 2).

Women aged between 36-45 who had one or more deliveries were 1.55-times less likely to be aware of HPV in comparison with women aged 18-25 who had one or more deliveries (Table 2). The subgroup analysis among women who had Pap test and HPV test showed different results in the awareness about HPV (Table 2). The following factors were positively associated with HPV awareness: age group of 36-45 (AOR=1.3[illegible]CI:0.78-2.42, referent=age group of 18-25), Russian ethnic group (AOR=2.11; CI:1.45-3.08, referent=Kazakh ethnic group), university degree (AOR=1.28; CI:0.83-1.97, referent= unfinished/finished school), having positive results of HPV test (AOR=1.63; CI:1.19-2.24, referent=negative results of HPV test). Factors negatively associated with HPV awareness were the following: college degree (AOR=0.90; CI:0.56-1.45, referent=unfinished/finished school), menarche after 15 (AOR=0.73; CI:0.42-1.26, referent=menarche before 13), having one or more deliveries (AOR=0.82; CI:0.54-1.25, referent=having no children), and having abnormal the Pap test result (AOR=0.47; CI:0.26-0.88[illegible]

The sources from which the study participants heard about HPV are shown in Fig 1. Majority (33.10%) of women heard about HPV from their gynecologist. The second most common source was the Internet (16.80%). The third and fourth most common sources of HPV information were general practitioner (7.40%) and television (6.90%). The rest of the sources

(5%) included other health professionals, friends and peers, family members, educational settings, magazines/books, and nurse practitioners.

Fig 1. Sources of information on HPV

HPV knowledge

Among those who were aware about HPV vaccine (N=1,215), less than half of the respondents (46.36%) knew that HPV is the major cause of cervical cancer (Table 3). Bivariate analysis using the chi-square test showed that there is statistically significant difference in the knowledge of HPV as a major cause of cervical cancer among ethnicity groups, education levels, and cities. In the multivariable logistic model, the following factors were positively associated with knowledge of HPV as the major cause of cervical cancer: age group of 26-35 (AOR=1.27; CI:0.80-1.85, referent=age group of 18-25), other ethnic group (AOR=1.11; CI:0.71-1.73, referent=Kazakh ethnic group), university degree (AOR=1.77; CI:1.24-2.52, referent= unfinished /finished school) and having one or more deliveries (AOR=1.14; CI:0.83-1.56, referent=having no delivery). A factor negatively associated with the knowledge of HPV as the major cause of cervical cancer was the Russian ethnic group (AOR=0.73; CI:0.55-0.99).

Table 3. Knowledge of HPV as the major cause of cervical cancer vaccination among women visiting gynecological clinics, (N=1126)

Variables	HPV is the major cause of cervical cancer		
	**N = 527; 46.36%<br>%, p value**	**OR (95% CI)<br>N=1126**	**AOR (95% CI)*<br>N=1125**
**Age**			
**18-25**	40.82	1	1
**26-35**	48.47	1.36 (0.93-1.99)	1.27 (0.85-1.88)
**36-45**	46.51	1.26 (0.85-1.86)	1.21 (0.80-1.85)
**46+**	45.71	1.22 (0.80-1.87)	1.23 (0.78-1.94)
	p-value = 0.444		
**Ethnicity**			
**Kazakh**	48.04	1	1
**Russian**	39.52	0.71 (0.53-0.94)	0.73 (0.55-0.99)

**Other**	50.56	1.11 (0.71-1.72)	1.11 (0.71-1.73)
	p-value = 0.045		
**Education**			
**Unfinished/finished school**	35.43	1	1
**College**	45.13	1.50 (1.03-2.18)	1.42 (0.98-2.08)
**University**	50.34	1.84 (1.30-2.62)	1.77 (1.24-2.52)
	p-value =0.002		
**Number of deliveries**			
**0**	43.70	1	1
**≥1**	47.13	1.15 (0.86-1.53)	1.14 (0.83-1.56)
	p-value = 0.346		

*Adjusted for age, ethnicity, education, number of deliveries

HPV vaccination

Among the study participants who were aware about HPV (N=1215), half of the respondents (51.53%) were also aware of HPV vaccine (Table 4). Bivariate analysis using chi-square test showed that there is statistically significant difference in HPV vaccine awareness within age groups, city groups, family groups, and menarche groups. In the multivariable logistic model, the following three factors were positively associated with HPV vaccine awareness: age group of 46 and older (AOR=1.91.; 95% CI:11.25-2.91, referent=age group of 18-25), Russian ethnic group (AOR=1.37; CI:0.99-1.83,), university degree (AOR=1..45; CI:1.03-2.04,), and having family or being in the relationship (AOR=1.41; CI:1.05-1.86,). A factor negatively associated with HPV vaccine awareness was menarche after 15 (AOR=0.50; CI:0.38-0.82, referent=menarche before 13).

Table 4. Awareness of HPV vaccination among women visiting gynecological clinics, (N=1207)

Variables	Aware of vaccine N = 633; 51.53% %, p value	OR (95% CI), N = 1207	AOR (95% CI)*, N = 207	AOR subgroup (95% CI)** N=1010
**Age,** mean 36.33±10.09				
**18-25**	42.50	1	1	1
**26-35**	48.67	1.28 (0.89-1.85)	1.17 (0.81-1.71)	1.10 (0.73-1.67)
**36-45**	55.16	1.66 (1.14-2.42)	1.59 (1.09-2.35)	1.47 (0.97-2.25)
**46+**	57.64	1.84 (1.22-2.77)	1.91 (1.25-2.91)	1.79 (1.12-2.86)
	p-value = 0.007			
**Ethnicity**				
**Kazakh**	49.28	1	1	1
**Russian**	57.09	1.37 (1.04-1.80)	1.37 (1.03-1.81)	1.35 (0.99-1.83)
**Other**	55.21	1.27 (0.83-1.94)	1.28 (0.83-1.98)	1.22 (0.77-1.94)
	p-value = 0.060			
**Education**				
**Unfinished/finished school**	47.51	1	1	1
**College**	48.20	1.03 (0.72-1.46)	1.06 (0.74-1.53)	1.20 (0.81-1.80)
**University**	54.70	1.33 (0.96-1.86)	1.45 (1.03-2.04)	1.44 (0.99-2.10)
	p-value = 0.065			
**Family**				
**Single**	44.80	1	1	1
**Not single**	53.29	1.41 (1.06-1.86)	1.41 (1.05-1.88)	1.31 (0.96-1.78)
	p-value = 0.017			
**Age of menarche,** mean 13.60±1.41				
**<13**	56.81	1	1	1
**13-15**	52.72	0.85 (0.63-1.15)	0.86 (0.63-1.18)	0.87 (0.63-1.22)
**>15**	43.10	0.58 (0.40-0.84)	0.50 (0.38-0.82)	0.52 (0.34-0.79)
	p-value = 0.008			

**Pap test status**				
**Normal**	52.36	1		1
**Abnormal**	40.00	0.61 (0.35-1.06)		0.56 (0.32-0.99)
	p-value = 0.075			

*Adjusted for age, ethnicity, education, family, age of menarche
**Adjusted for age, ethnicity, education, family, age of menarche, Pap test status

In the multivariable logistic model of a subgroup (N=1010), the following three factors were positively associated with HPV vaccine awareness: age group of 46 and older (AOR=1.7[illegible] CI:1.12-2.86, referent=age group of 18-25), Russian ethnic group (AOR=1.35; CI:0.99-1.83, referent=Kazakh ethnic group), university degree (AOR=1.44; CI:0.99-2.10, referent=unfinished/finished school), and having family or being in a relationship (AOR=1.31; CI:0.96-1.78, referent=being single). Factor negatively associated with HPV vaccine awareness was menarche after 15 (AOR=0.50; CI:0.38-0.82, referent=menarche before 13), and the following three factors were positively associated with HPV vaccine awareness: age group of 46 and older (AOR=1.9[illegible] CI:11.25-2.91, referent=age group of 18-25), Russian ethnic group (AOR=1.37; CI:0.99-1.83,), university degree (AOR=1.45; CI:1.03-2.0[illegible] and having family or being in a relationship (AOR=1.41; CI:1.05-1.86). Two factors negatively associated with HPV vaccine awareness were menarche after 15 (AOR=0.52; CI:0.34-0.79, referent= menarche before 13) and having abnormal Pap test result (AOR=0.56; CI:0.32-0.99, referent=having normal result from the Pap test).

Discussion

This is the first study in Kazakhstan was aimed to assess the knowledge and awareness of HPV as a cause of cervical cancer, and the awareness of HPV vaccination among 18-70-year-old women visiting gynecological clinics in five different regions of Kazakhstan. Also, this study aimed to identify the factors associated with the awareness of HPV and HPV vaccine, and

knowledge of HPV as a major cause of cervical cancer. Cervical cancer causes over 300 000 deaths per year worldwide and is the second leading cause of death from cancer among women in Kazakhstan. It is important to implement prevention strategies to reduce spread of the disease. The major cause of cervical cancer is HPV infection. It has been demonstrated that the HPV vaccine prevents cervical cancer. Therefore, information about not only HPV, but also HPV vaccination are important topics to study as it can help in the prevention of cervical cancer.

In this prospective cross-section[illegible]tudy more than half of the participants (N=2281, 53.43%) were aware about HPV infection. The observed level of HPV awareness is similar to the results of a study conducted in Serbia, where cervical cancer is one of the most frequent type of cancers. The results of the study conducted among women visiting Serbian cervical cancer counseling center showed 60.5% of HPV awareness [20]. The results of HPV awareness from this study are also similar to the results from the studies conducted in other high middle-income countries. The study in Brazil showed that there is 40% of awareness of HPV, and the study conducted in China showed a 51.1% prevalence of HPV awareness [21,22].

In our study, the major sources of information about HPV vaccine were gynecologists and general practitioners, the Internet and TV. Results from other studies on HPV awareness confirm that medical workers and media are the main source of awareness about HPV and cervical cancer [21,22]. Such relatively high prevalence of HPV awareness can be attributed to the fact that healthcare in Kazakhstan is free, and therefore there are fewer barriers for women to visit medical facilities.

Our investigation revealed that only 46.36% of women were aware that HPV is the major cause of cervical cancer among those who were aware of HPV. Results of our study are similar to the stud[illegible]nducted in China where knowledge of HPV as a cause of cervical cancer is 39.6% [22]. It is important to note that awareness about HPV was relatively higher than the knowledge

of HPV. Therefore, high level of awareness of HPV does not necessarily lead to a high level of knowledge of HPV. It is also important to note that among total number of participants the knowledge of HPV is much lower, around 23.1% (527 out of 2,281 women). Among those who had heard about HPV infection, half of the respondents (51.53%) of this study were aware about HPV vaccine. Among all recruited women, awareness about HPV vaccine is only about 27.75% (633 out of 2,281 women). Such relatively low level of vaccine awareness is a common trend in other countries as well. In Serbia there was only a 23% of HPV vaccine awareness, in China only 15.6% of women were aware of HPV vaccine [20,22].

One of the most important findings of this study is that having a university degree is positively associated with HPV awareness, knowledge of HPV as the major cause of cervical cancer, and awareness of the HPV vaccine. This finding is similar to the results of the study conducted in the US, which found that adults with college degrees are more likely to be aware and knowledgeable about HPV and HPV vaccination [23]. People that had a university degree in this study were women that had a bachelor's degree, Master’s degree, or Ph.D. degree. Therefore, this group included women who had dedicated more time of their lives for education in comparison with those who completed just school or college.

Older age was also a positive factor for all outcome variables in this study. Women who were aged 46 or older were more likely to be aware of HPV and HPV vaccine. However, women in the age group of 26-35 were more likely to be more knowledgeable about HPV as the major cause of cervical cancer. Both of these age groups include the age that is included in the compulsory cervical screening, between 30 and 70 [13]. Therefore, as these women tend to visit the gynecologists at least once in four years for cervical screening, they are more likely to have access to information about HPV and HPV vaccine.

This study also found ethnicity as a positive factor for all three outcome variables. Women from ethnic groups other than Kazakh or Russian were more likely to be aware of HPV and know that HPV causes cervical cancer. Women from the Russian ethnic group were more likely to be aware of the HPV vaccine. Findings regarding ethnic groups demonstrate that Kazakh women are less aware of HPV and HPV vaccination. This might be explained by the fact that the majority of the sources of information about HPV and HPV vaccination are communicated in the Russian language. Therefore, women who can only speak Kazakh have limited access to this information.

Having one or more deliveries was found to be a positive factor for HPV knowledge. This can be explained by the fact that women having the deliveries were more likely to visit the hospital and while there, they will get information about HPV infection. In the case of HPV awareness, having one or more deliveries was found to be a negative factor. However, an interaction was found between age and delivery in the model of HPV awareness. Women in the age group of 36-45 who had one or more deliveries were more likely to be aware of HPV in comparison with women aged 18-25 who also had one or more deliveries. Therefore, women who were older and had one or more deliveries were more likely to be aware of HPV in comparison with younger women.

Regarding awareness of the HPV vaccine, having a family or being in a committed relationship was found to be a positive factor. Women who were not single were more likely to be aware of the HPV vaccine. This can be attributed to the fact that women who were married or in a relationship were more likely to have a child and therefore visit the hospital where they could get the information about HPV vaccination.

Having menarche after 15 years of age was found to be a negative factor for all three outcome variables. Studies showed that girls having menarche in earlier ages before 12 tend to be

interested in sexual life at earlier ages [24]. Therefore, women who had menarche after 15 were less likely to have sexual relations at an earlier age; as a result, they were less aware of sexually transmitted infections such as HPV.

Subgroup analysis of women that had HPV and Pap smear tests showed that HPV test with a positive result is a positive factor for HPV awareness and Pap smear with an abnormal result is a negative factor for HPV awareness. Pap smear test with an abnormal result was also a negative factor for the awareness of HPV vaccination. Pap smear test is not a direct test to detect HPV, but rather is a test to check the smear for abnormal cytology o cervical cells; therefore, women who had Pap smear testing were not necessarily aware of HPV and HPV vaccination. However, undergoing an HPV test means that women will at least hear about the HPV word itself thus may be curious to learn more about the virus and why this test was conducted.

This is the first study to evaluate the level of knowledge and awareness of women in Kazakhstan on the topics of HPV and HPV vaccination, which covers all the country's regions. The study demonstrates a lack of knowledge among the public where half of the women were aware of HPV infection, only a quarter knew of the link between HPV and cervical cancer, and only a quarter of interviewed women were aware of the vaccine against HPV.

Strength and limitations

Due to the design of our study being an observational cross-sectional study, no causality can be established between the chosen factors and level of knowledge and awareness of HPV and HPV vaccination. Moreover, the convenient sampling method used in this study introduced selection bias. We did not have socio-demographic characteristics of women who did not participate in our study but also attended gynecological clinics, so we cannot determine whether there is a significant difference between respondents and non-respondents. Considering the self-

reporting nature of the participants' answers, this could also result in recall bias, underreporting or exaggeration of the responses.

Although our study had more than two thousand participants from the north, south, east, west, and center of the country, the results of our study might not be applicable to all women in Kazakhstan. Sampling through convenience in gynecological offices could exclude certain demographic groups. Moreover, despite a big movement of people from rural parts of the country to the cities, rural areas of the country can differ significantly from the cities where the study took place. Therefore, a sizable portion of the female population is not represented in the study.

Clinical implication

To decrease the rate of cervical cancer in Kazakhstan all existing technologies such as HPV infection testing, effective cervical cancer screening, colposcopy examination, and vaccination should be employed. However, the success of the governmental programs for cervical cancer prevention will depend on the population's knowledge and awareness of the problem and the ways of prophylactics. In this view, population-based information programs to enhance the knowledge and awareness, thus increasing understanding of the risks related to HPV infection and its association with cervical cancer could enhance the public's acceptance of the screening program. The screening program should be more carefully implemented and reinforced to reach a demand level of 70% [1].

HPV vaccination should be initiated again after care[illegible] education of the population and proper introduction. Before introducing HPV vaccine into the national immunization schedule and to increase possible vaccination uptake, it is crucial to conduct research to better understand the population's knowledge, awareness, and understanding barriers to vaccination, attitudes, practices, and beliefs about immunization specifically regarding the HPV vaccine. The task

appears even more complex due to the challenges faced during the previous HPV vaccination effort in Kazakhstan, along with the current public hesitancy towards vaccines in general. The overall goal is to work toward informing the population in terms of best practices that should be adopted with regards to screening and vaccination prevention programs. This would result in a significant reduction in the incidence and prevalence of cervical cancer in Kazakhstan and will help the country to achieve the goals set by the WHO.

Conclusions

This cross-sectional study suggests that among 2,281 women visiting gynecological clinics in Kazakhstan the awareness of HPV is relatively high (53%). Although among women who aware of HPV the knowledge that HPV causes cervical cancer and HPV vaccination is around 50% each, there is relatively low (around 25% each) prevalence among total number of participants of the study. Outcome variables are associated with age, ethnicity, education, family and gynecological characteristics such as menarche, delivery, HPV test, and Pap smear test[illegible]s the success of the governmental programs for cervical cancer prevention depends on the population's knowledge and awareness of HPV and cervical cancer, there is an urgent need for educational intervention; both formal and informal. Population-based information programs need to enhance the knowledge and awareness, which would increase understanding of the risks related to HPV infection and its association with cervical cancer. Overall, this could enhance the public's acceptance of the HPV screening program for cervical cancer prevention.

Acknowledgements

The author would like to acknowledge the Nazarbayev University School of Medicine for the supportive atmosphere that enabled completion on this study.

References

1. World Health Organization. Human papillomavirus (HPV) and cervical cancer [Internet]. 2020. Available from: https://www.who.int/news-room/fact-sheets/detail/human-papillomavirus-(hpv)-and-cervical-cancer
2. Chesson HW, Dunne EF, Hariri S, Markowitz LE. The estimated lifetime probability of acquiring human papillomavirus in the United States. Sex Transm Dis. 2014;41(11):660–4.
3. Raychaudhuri S, Mandal S. Current status of knowledge, attitude and practice (KAP) and screening for cervical cancer in countries at different levels of development. Asian Pac J Cancer Prev [Internet]. 2012;13(9):4221–7. Available from: http://www.ncbi.nlm.nih.gov/pubmed/23167318
4. Cohen PA, Jhingran A, Oaknin A, Denny L. Cervical cancer. Lancet [Internet]. 2019;393(10167):169–82. Available from: http://dx.doi.org/10.1016/S0140-6736(18)32470-X
5. Arbyn M, Weiderpass E, Bruni L, de Sanjosé S, Saraiya M, Ferlay J, et al. Estimates of incidence and mortality of cervical cancer in 2018: a worldwide analysis. Lancet Glob Heal. 2020;8(2):e191–203.
6. World Health Organization. A cervical cancer-free future: First-ever global commitment to eliminate a cancer [Internet]. 2020 [cited 2021 Feb 17]. Available from: https://www.who.int/news/item/17-11-2020-a-cervical-cancer-free-future-first-ever-global-commitment-to-eliminate-a-cancer
7. Canfell K. Towards the global elimination of cervical cancer. Papillomavirus Res [Internet]. 2019;8(June):100170. Available from: https://doi.org/10.1016/j.pvr.2019.100170
8. Zhumadilova A, Craig BJ, Bobak M. Patient-centered beliefs among patients and providers in Kazakhstan. Ochsner J. 2018;18(1):46–52.
9. Bekmukhambetov YZ, Balmagambetova SK, Jarkenov TA, Nurtayeva SM, Mukashev TZ, Koyshybaev AK. Distribution of high risk human papillomavirus types in Western Kazakhstan - retrospective analysis of PCR data. Asian Pacific J Cancer Prev. 2016;17(5):2667–72.
10. Niyazmetova L, Aimagambetova G, Stambekova N, Abugalieva Z, Seksembayeva K, Ali S, et al. Application of molecular genotyping to determine prevalence of HPV strains in Pap smears of Kazakhstan women. Int J Infect Dis [Internet]. 2017;54:85–8. Available from: http://dx.doi.org/10.1016/j.ijid.2016.11.410
11. Bruni L, Albero G, Serrano B, Mena M, Gómez D, Muñoz J, Bosch FX de SS. Human Papillomavirus and Related Diseases in Kazakhstan. Summary Report. Inf Cent HPVand Cancer (HPV Inf Centre) [Internet]. 2019;(June). Available from: www.hpvcentre.com
12. Aimagambetova G, Chan CK, Ukybassova T, Imankulova B, Balykov A, Kongrtay K, et al. Cervical cancer screening and prevention in Kazakhstan and Central Asia. J Med Screen. 2020;4–6.
13. Kaidarova D. The experience of Cervical Cancer screening program in Kazakhstan. In: Eurasian Cancer Screening Conference. Minsk; 2018.
14. Kaidarova, D., Zhylkaidarova, A., Dushimova, Z., Bolatbekova R. Screening for Cervical Cancer in Kazakhstan. J Glob Oncol [Internet]. 2018;4. Available from: https://ascopubs.org/doi/abs/10.1200/jgo.18.65600

15. Bekmukhambetov Y, Mynbaev O, Tinelli A, Balmagambetova S, Koyshybaev A, Urazayev O, et al. Human Papillomavirus related issues in western Kazakhstan: Protocol for a comprehensive study. Russ Open Med J [Internet]. 2018;7(4). Available from: http://www.embase.com/search/results?subaction=viewrecord&from=export&id=L625491252%0Ahttp://dx.doi.org/10.15275/rusomj.2018.0408
16. Dany M, Chidiac A, Nassar AH. Human papillomavirus vaccination: Assessing knowledge, attitudes, and intentions of college female students in Lebanon, a developing country. Vaccine [Internet]. 2015;33(8):1001–7. Available from: http://dx.doi.org/10.1016/j.vaccine.2015.01.009
17. Pitts M, Clarke T. Human papillomavirus infections and risks of cervical cancer: What do women know? Health Educ Res. 2002;17(6):706–14.
18. Waller J, McCaffery K, Forrest S, Szarewski A, Cadman L, Wardle J. Awareness of human papillomavirus among women attending a well woman clinic. Sex Transm Infect. 2003;79(4):320–2.
19. StataCorp LLC. Stata Statistical Software: Release 16 [Internet]. 2019. Available from: https://www.stata.com/support/faqs/resources/citing-software-documentation-faqs/#:~:text=2019.,Station%2C TX%3A StataCorp LLC.&text=StataCorp.,-2017.
20. Djuric O, Markovic-Denic L, Popovac S, Todorovic J, Marusic V, Maksimovic N. Awareness and knowledge about HPV infection and HPV vaccination among women undergoing cytology and colposcopy in Serbian cervical cancer counseling center. J BUON. 2020;25(1):116–24.
21. Osis MJD, Duarte GA, de Sousa MH. SUS users' knowledge of and attitude to HPV virus and vaccines available in Brazil. Rev Saude Publica. 2014;48(1):123–33.
22. Yuanyue L, Baloch Z, Shanshan L, Yasmeen N, Xiaomei W, Khan JM, et al. Cervical Cancer, Human Papillomavirus Infection, and Vaccine-Related Knowledge: Awareness in Chinese Women. Cancer Control. 2018;25(1):1–8.
23. Thompson EL, Wheldon CW, Rosen BL, Maness SB, Kasting ML, Massey PM. Awareness and knowledge of HPV and HPV vaccination among adults ages 27–45 years. Vaccine [Internet]. 2020;38(15):3143–8. Available from: https://doi.org/10.1016/j.vaccine.2020.01.053
24. Yoo J. Effects of early menarche on physical and psy - chosocial health problems in adolescent girls and adult women. 2016;59(9):355–61.

Figure 1 Click here to access/download;Figure;Fig1.tiff.tiff

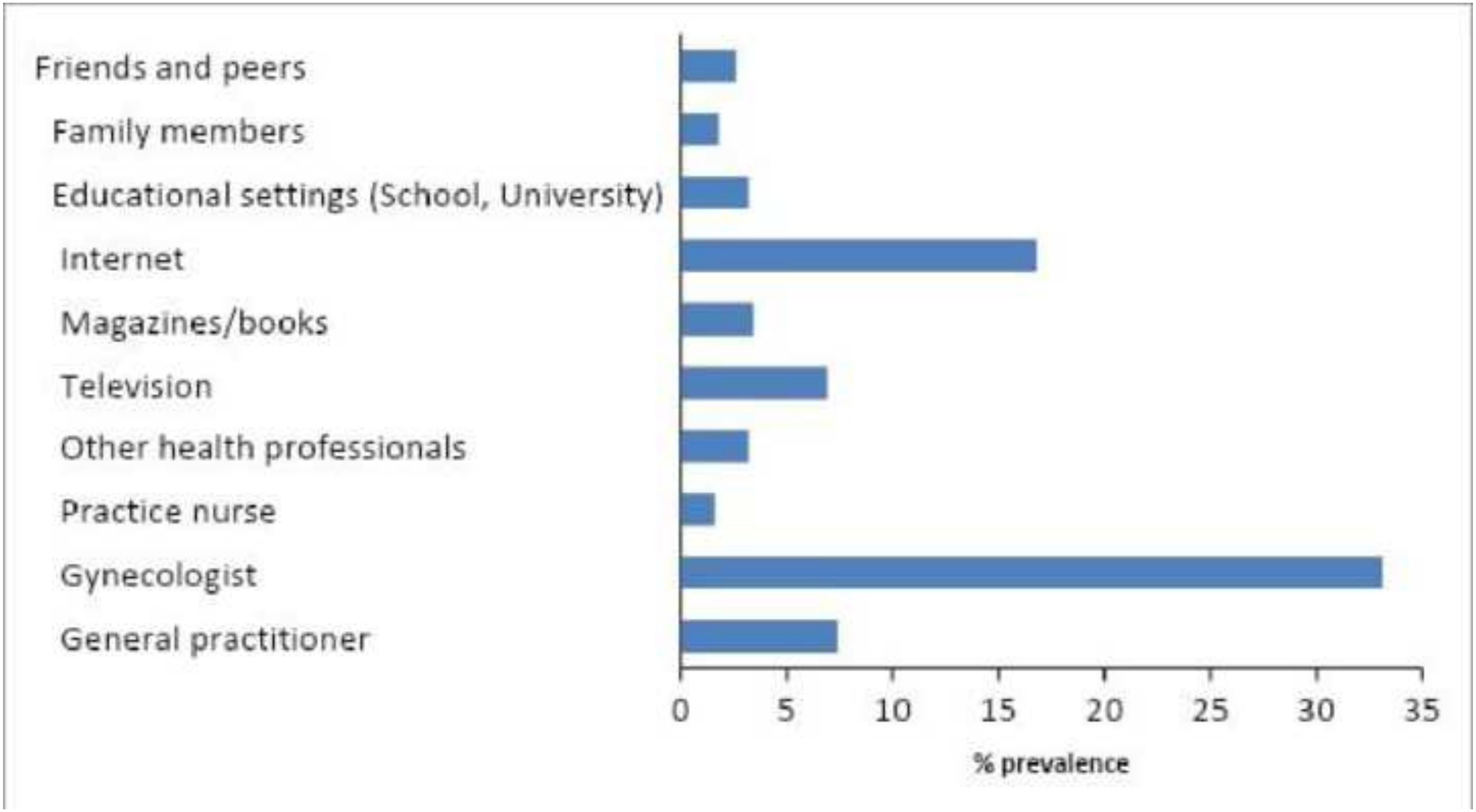

Supplement: Attachment — Submitted filename: PONE-D-21-11756_MM_august 2020.pdf [file pone.0261203.s004.pdf]
