# Supplementary material for: Knowledge and awareness of human papillomavirus infection and human papillomavirus vaccine among Kazakhstani women attending gynecological clinics
Source: PLoS One. 2021 Dec 13;16(12):e0261203. doi: 10.1371/journal.pone.0261203 (PMC8668105; doi:10.1371/journal.pone.0261203)
Supplement: Attachment — Submitted filename: Response to the reviewers.docx [file pone.0261203.s005.docx]

**Dear Editorial Team,**

**Thank you very much for the careful evaluation of our manuscript. We appreciate your time, efforts, valuable comments and suggestions that helped us to improve the manuscript quality. Please find below our point-by-point responses for all your comments.**

Journal Requirements:

When submitting your revision, we need you to address these additional requirements.

If applicable, we recommend that you deposit your laboratory protocols in [protocols.io](http://protocols.io/) to enhance the reproducibility of your results. Protocols.io assigns your protocol its own identifier (DOI) so that it can be cited independently in the future. For instructions see: <https://journals.plos.org/plosone/s/submission-guidelines#loc-laboratory-protocols>.

**Response:** According to the recommendations, the survey protocol is now deposited on protocols.io and could be reached via the link: <https://www.protocols.io/workspaces/medicine1/resources/knowledge-and-awareness-of-human-papillomavirus-infection>

In addition, all study questionnaires as well as the raw data are available via the link:

https://zenodo.org/record/4600664#.YHBChs9xfIV

1. Please ensure that your manuscript meets PLOS ONE's style requirements, including those for file naming. The PLOS ONE style templates can be found at
   <https://journals.plos.org/plosone/s/file?id=wjVg/PLOSOne_formatting_sample_main_body.pdf> and 
   <https://journals.plos.org/plosone/s/file?id=ba62/PLOSOne_formatting_sample_title_authors_affiliations.pdf>

**Response:** The text of the manuscript has been corrected according to the journal requirements.

2. Please review your reference list to ensure that it is complete and correct. If you have cited papers that have been retracted, please include the rationale for doing so in the manuscript text, or remove these references and replace them with relevant current references. Any changes to the reference list should be mentioned in the rebuttal letter that accompanies your revised manuscript. If you need to cite a retracted article, indicate the article’s retracted status in the References list and also include a citation and full reference for the retraction notice.

**Response:** The list of references related to this manuscript has been carefully reviewed by all authors. We do not cite any retracted reference. All references we are citing in this manuscript can be found in Pub Med.

3. We suggest you thoroughly copyedit your manuscript for language usage, spelling, and grammar. If you do not know anyone who can help you do this, you may wish to consider employing a professional scientific editing service.

Whilst you may use any professional scientific editing service of your choice, PLOS has partnered with both American Journal Experts (AJE) and Editage to provide discounted services to PLOS authors. Both organizations have experience helping authors meet PLOS guidelines and can provide language editing, translation, manuscript formatting, and figure formatting to ensure your manuscript meets our submission guidelines. To take advantage of our partnership with AJE, visit the AJE website (<http://learn.aje.com/plos/>) for a 15% discount off AJE services. To take advantage of our partnership with Editage, visit the Editage website ([www.editage.com](http://www.editage.com/)) and enter referral code PLOSEDIT for a 15% discount off Editage services.  If the PLOS editorial team finds any language issues in text that either AJE or Editage has edited, the service provider will re-edit the text for free.

Upon resubmission, please provide the following:

The name of the colleague or the details of the professional service that edited your manuscript

A copy of your manuscript showing your changes by either highlighting them or using track changes (uploaded as a *supporting information* file)

A clean copy of the edited manuscript (uploaded as the new *manuscript* file)”

**Response:** Unfortunately, our budget is very limited, and we have no resources to pay and use editing service.

The thorough English editing was done by prof. Azliyati Azizan, professor of Touro University, Nevada, USA.

The edited manuscript are submitted in track changes and clean versions.

4. Please include additional information regarding the survey or questionnaire used in the study and ensure that you have provided sufficient details that others could replicate the analyses. For instance, if you developed a questionnaire as part of this study and it is not under a copyright more restrictive than CC-BY, please include a copy, in both the original language and English, as Supporting Information.

**Response:** There are two questionnaires used in the study. Both were developed by the team members and submitted as Supplementary information to the manuscript in English and local languages (Kazakh and Russian). Anyone who would like to reproduce this study are welcome to use our protocol and questionnaires.

In your Methods section, please provide a justification for the sample size used in your study, including any relevant power calculations (if applicable).

**Response:** The sample size was calculated. We used data for exposed and unexposed groups from the following study: Yuanyue L, Baloch Z, Shanshan L, Yasmeen N, Xiaomei W, Khan JM, et al. Cervical Cancer, Human Papillomavirus Infection, and Vaccine-Related Knowledge: Awareness in Chinese Women. Cancer Control. 2018;25(1):1–8.

Exposed (those who are aware of HPV as a cause of cervical cancer)-40%

Unexposed (those who are not aware of HPV as a cause of cervical cancer)-60%

According to calculation in Epi Info : calculated sample size is 223.

However, we have gathered many more. In 2019 and in 2020 we obtained approximately 200 questionnaires from each city each year. Overall it resulted in more than 2000 patients’ surveys.

Finally please provide additional information regarding further treatment for women whom where identified as HPV positive during the study (for instane whether they were referred to a medical specialist for further treatment upon knowledge of their diagnosis).

**Response:** All HPV positive women were invited for follow-up consultation and managed according to the national guideline or cervical lesions management.

5. Thank you for stating the following financial disclosure: 
 "This study was supported by the Faculty Development Research Grant Program 2019-2021 (Funder Project Reference: 110119FD4528, title: A molecular epidemiological study to determine the prevalence of oncogenic HPV strains for CC prevention in Kazakhstan). The funder had no role in study design, data collection and analysis, decision to publish, or preparation of the manuscript. GA. is a PI of the project."

We note that one or more of the authors is affiliated with the funding organization, indicating the funder may have had some role in the design, data collection, analysis or preparation of your manuscript for publication; in other words, the funder played an indirect role through the participation of the co-authors. If the funding organization did not play a role in the study design, data collection and analysis, decision to publish, or preparation of the manuscript and only provided financial support in the form of authors' salaries and/or research materials, please do the following:

a. Review your statements relating to the author contributions, and ensure you have specifically and accurately indicated the role(s) that these authors had in your study. These amendments should be made in the online form.

**Response:** The statements relating to the author contributions were reviewed and the authors’ contributions were accurately indicated.

b. Confirm in your cover letter that you agree with the following statement, and we will change the online submission form on your behalf: 
“The funder provided support in the form of salaries for authors [insert relevant initials], but did not have any additional role in the study design, data collection and analysis, decision to publish, or preparation of the manuscript. The specific roles of these authors are articulated in the ‘author contributions’ section.

**Response:** I confirm, “The funder provided support in the form of salaries for authors T.I. and A.B., but did not have any additional role in the study design, data collection and analysis, decision to publish, or preparation of the manuscript. The specific roles of these authors are articulated in the ‘author contributions’ section.”

**Response to the Reviewers**

**Dear Reviewer,**

**Thank you very much for the careful evaluation of our manuscript. We appreciate your time, efforts, valuable comments and suggestions that helped us to improve the manuscript quality. Please find below our point-by-point responses for all your comments.**

Reviewer #1: This is a very good manuscript. A very interesting and actual topic, which was very clear and well presented by the authors. The study design and statistical analysis are appropriate. The refferences are very well chosed.

However, there are some minor issues that have to be addressed.
1. There are some minor ortographic erors (highlighted in the reviewed manuscript)

**Response:** Thank you very much for the comment. A careful editing of the manuscript have been performed. Errors and typos are removed. Please see the manuscript submitted with the track changes.

2. In the tables is better to present the results as: numbers (and percentage) in order to be more explicit and to have a more real image of the values. For example: 10 (3.25).

**Response:** Thank you for the comment. All table were recomposed and the results are presented in numbers (and percentages) as was suggested. Please see the manuscript submitted with the track changes.

3. A cross-sectional study CAN NOT BE a prospective study!!!!!! A cross-sectional study means like "a snapshot".
A prospective study involves following up the subjects over a period of time (longitudinal collection of data).

**Response**: Thank you or the comment. The error was removed and the sentence now appears as follows: “A ~~prospective~~ cross-sectional study among women from five cities of central (Nur-Sultan, the capital city), southern (Almaty), western (Aktobe), northern (Pavlodar), and eastern (Oskemen) parts of Kazakhstan was conducted from May 25, 2019, until December 2020.”

4. Usually in the "Conclusions" section the authors do not present figures and percentage form the "Results/ Discussions" sections. They present the general conclusions of the study, which were very well highlighted for the current research below the phrase with the figures, in the Conclusions section of the manuscript.

**Response:** Thank you for the comment. The conclusion section was recomposed as was suggested and now appears as follows: “The results of this cross-sectional study suggests that the awareness of HPV among women visiting gynecological clinics in Kazakhstan is relatively high. However, the knowledge that HPV causes cervical cancer and awareness of HPV vaccination should be improved. As the success of the governmental programs for cervical cancer prevention largely depends on the population's knowledge and awareness of HPV and cervical cancer, there is an urgent need for educational intervention; both formal and informal. Population-based informational programs need to enhance the knowledge and awareness, which would increase understanding of the risks related to the HPV infection and its association with cervical cancer. Overall, this could improve the public's acceptance of the cervical cancer screening program in Kazakhstan.”

**Dear Reviewer,**

**Thank you very much for the careful evaluation of our manuscript. We appreciate your time, efforts, valuable comments and suggestions that helped us to improve the manuscript quality. Please find below our point-by-point responses for all your comments.**

Reviewer #2: Knowledge and awareness of HPV infection and HPV vaccine among Kazakhstani women
Issa et al present the findings from the well done study “Kazakhstani women’s Knowledge and awareness of HPV infection and HPV vaccine.”

The tables and figure are clear and concise.

The text, in contrast, especially in RESULTS is difficult to read and needs to be simplified. For example, is it necessary to repeat the data from the tables, especially that contained in parentheses (e.g., AOR=0.73.; CI: 0.39-1.34, referent=age group of 18-25).
L217: The text states that women age 46 and over were aware of HPV, which I take to mean this is an important finding. To me it then implies that younger women likely are not. Is it then necessary several lines later to actually state that younger women were not.
**Response**: Thank you very much for the comment. We are very sorry for the issues with the test. For non-native English speakers it is challenging. The manuscript text has been reviewed by the coauthor, Professor from Touro University, USA, Azliyati Azizan. The text was made more simple and clear. The results section was simplified.

L235-L240 The text repeats data in results. The entire RESULTS section should be made more readable

**Response:** The results section was recomposed and repetitions in the text and tables were removed.


L243: (Among those who were aware about HPV vaccine (N=1,215), less than half of the respondents (46.36%) knew that HPV is the major cause of cervical cancer).
If I interpret the authors’ meaning correctly, fewer than half of the 1215 women who were aware of the vaccine understood what the vaccine treated because they were not aware that HPV is the cause of cervical cancer. If so, the implication the implication for the need for education is important: What is cancer and how does it come about; what are vaccines and what do they treat.

**Response:** Thank you very much for the comment. We agree with the reviewer that the educational intervention is of a great importance and we highlighted it in the conclusion.

L297-L304: Largely repeats the introduction. It should be truncated or eliminated. Much of the remaining DISCUSSION unnecessarily repeats the results and can be truncated.

**Response:** Thank you for the comment. The discussion part was revised to eliminate repetitions between the discussion and the introduction and results parts. Please see the manuscript submitted with the track changes.

The CONCLUSION section repeats too much of the results, but insufficiently says what was learned from the study.

**Response:** Thank you for the comment. The conclusion section was recomposed as was suggested and now appears as follows: “The results of this cross-sectional study suggests that the awareness of HPV among women visiting gynecological clinics in Kazakhstan is relatively high. However, the knowledge that HPV causes cervical cancer and awareness of HPV vaccination should be improved. As the success of the governmental programs for cervical cancer prevention largely depends on the population's knowledge and awareness of HPV and cervical cancer, there is an urgent need for educational intervention; both formal and informal. Population-based informational programs need to enhance the knowledge and awareness, which would increase understanding of the risks related to the HPV infection and its association with cervical cancer. Overall, this could improve the public's acceptance of the cervical cancer screening program in Kazakhstan.”
